# Extraction of giant bladder calcium oxalate stone: A case report

**DOI:** 10.1016/j.ijscr.2020.02.055

**Published:** 2020-02-28

**Authors:** Navin Shrestha, Le Zhou, Chun Huan Hu

**Affiliations:** Wuhan Jingdu Lithiasis Urology Hospital, Hubei Province, Wuhan City, Wuchang District, Youyi Avenue, Caihua Street 1, 430063, PR China

**Keywords:** Bladder stone, Cystolithotomy, Calcium–oxalate

## Abstract

**Introduction:**

Bladder stone is a rare and ancient disease. Nowadays new technologies have been developed in the effort to make less invasive stone treatment. Bladder calculi account for 5% of urinary calculi.

**Presentation of case:**

A 52-year-old male patient with symptoms of lower abdominal pain, dysuria and pollakiuria was admitted. Urinalysis showed that pH5.0 and presence of calcium oxalate crystals and leukocyturia but erthrocyturia and nitrite were absent. Abdominal ultrasonogry revealed hydronephrosis, thickened bladder wall and large single stone. Plain radiography showed a large bladder stone measuring 12 × 10 cm.

**Discussion:**

In our case 1 extremely large bladder calculus occupied most of the bladder and pressing on the orifices of the ureters, leading to the presence of hydronephrosis. For large-sized bladder stones, all the reports have recommended open cystolithotomy.

**Conclusion:**

The combination of improved nutrition and modern antibiotic treatment has to be led to the frequency of bladder lithiasis. Calcium intake shouldn’t be restricted, whereas oxalate, sodium, and protein intakes have to be limited.

## Highlights

• Hyperoxaluria, hypercalciuria and a low urine calcium–oxalate ratio are involved in calcium oxalate monohydrate stone.
• For large-sized bladder stones, all the reports have recommended open cystolithotomy.
• Hyperoxaluria, and low urinary pH may promote the stone formation.
• Containment of animal protein and salt can reduce the relative risk of stone.

## 1 Introduction

Bladder stone is a rare and ancient disease. Nowadays new technologies have been developed in the effort to make less invasive stone treatment. Bladder calculi account for 5% of urinary calculi [1] and usually occur because of bladder outlet obstruction, neurogenic voiding dysfunction, infection, or foreign bodies. Patients usually have other lithogenous factors such as low urinary pH, low urinary magnesium and increased urinary uric acid super saturation [2]. Hyperoxaluria, hypercalciuria and a low urine calcium–oxalate ratio are involved in calcium oxalate monohydrate urinary stone formation [3].

Unfortunately, few contemporary series regarding bladder calculi exist in the worldwide literature. The association between bladder stone and urinary stasis is not entirely clear. Unlike studies of upper urinary tract stone disease, factors contributing to the pathogenesis of bladder calculi have not been well explored [4]. In recent years, bladder stones are increasing in China. However, a giant bladder stone is rarely found nowadays. The work has been reported in line with the SCARE criteria [5].

## 2 Case presentation

**Fig. 1:**
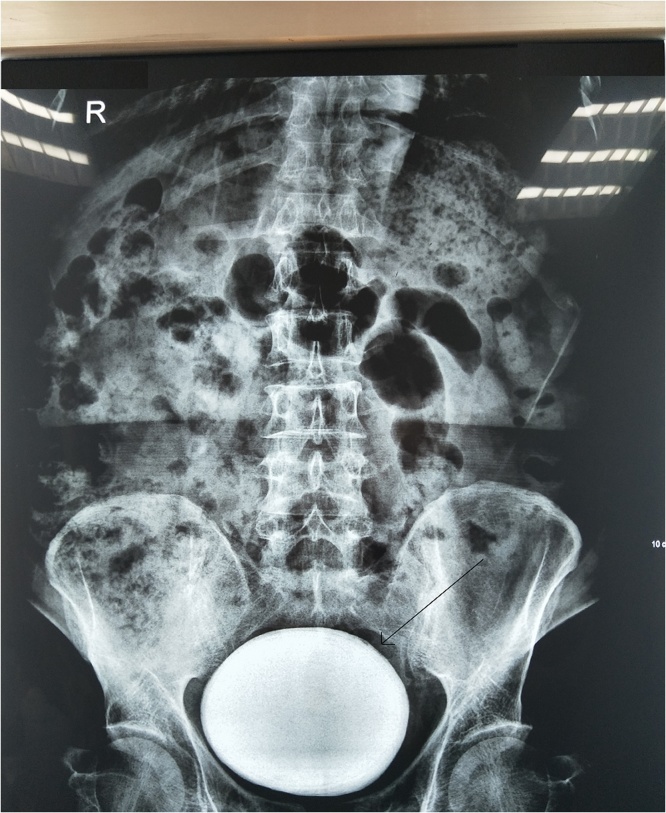
X-ray film showing giant, regular bladder stone.

A 52-year-old male patient with symptoms of lower abdominal pain, dysuria and pollakiuria was admitted to Urology department of our Hospital. The patient did not have gross hematuria, no past surgical history and other disease. The patient had some antibiotics for urinary tract infection for the past 2 year. Physical examination revealed severe tenderness in lower abdomen. Vital signs were normal. Digital rectal examination revealed normal prostate. Urinalysis showed that pH5.0 and presence of calcium oxalate crystals and leukocyturia but erthrocyturia and nitrite were absent. Routine hemogram was normal. Blood urea nitrogen, serum creatinine level and uric acid level were 4.17 mmol/L,167 μmol/L, 59.7 μmol/L respectively. Kidney and liver function were normal. Abdominal ultrasonogry revealed hydronephrosis, thickened bladder wall and large single stone. Plain radiography showed a large bladder stone measuring 12 × 10 cm (Fig. 1). 24-h urine specimen analysis showed that calcium was 7.1 mmol/day and oxalate was 0.62 mmol/day.

**Fig. 2:**
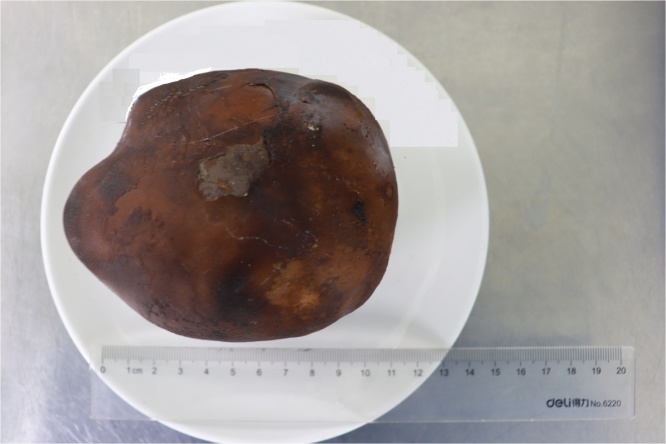
Giant bladder stone measuring 12.8 × 9.2 × 7.2 cm.

Patient underwent open cystolithotomy under general anesthesia and larger bladder stone was removed. The bladder stone weighed 950 g and measured 12.8 × 9.2 × 7.2 cm (Fig. 2). Stone analysis showed stone composed of 91% calcium oxalate monohydrate and 9% oxalate.

Hydronephrosis recovered to normal level within 5days. Patient decreased the consumption of oxalate-rich food. Patient was discharged on day 8. At 3 months of follow-up, the patients renal function was normal and voiding dysfunction disappeared.

## 3 Discussion

Bladder calculi account for 5% of urinary calculi and usually occur because of bladder outlet obstruction, neurogenic voiding dysfunction, infection, or foreign bodies [5]. Super saturation and crystallization are the main drivers for the etiopathogenesis of uric acid, xanthine and cystine stones but this physiochemical concepts fails to adequately explain the formation of calcium-based nephrolithiasis, which represents the majority of kidney stones [6]. The composition of bladder calculus is influenced by the pH and the degree of saturation of the urine. Children remain at high risk for developing bladder lithiasis in endemic areas. Males with prostate disease or relevant surgery and women who undergo anti-incontinence surgery are at a higher risk for developing vesical lithiasis. Open surgery remains the main treatment of bladder calculus in children. In adults, the classical treatment for bladder calculi is endoscopic transurethral disintegration with mechanical cystolithotripsy, ultrasound, electrohydraulic lithotripsy, Swiss Lithoclast, and holmium:YAG laser [7].

In our case 1 extremely large bladder calculus occupied most of the bladder and pressing on the orifices of the ureters, leading to the presence of hydronephrosis. For large-sized bladder stones, all the reports have recommended open cystolithotomy [8]. Calcium oxalate stone development is relatively slow and may cause clinical symptoms and be removed before growing to a large size [9]. In our case, hyperoxaluria, and low urinary pH may promote the stone formation. The dietary habits of the patient were responsible to the hyperoxaluria, as he tend to eat oxalate-rich foods. A diet based on a adequate intake of calcium (1000–2000 mg per day) and containment of animal protein and salt can decrease significantly urinary super saturation for calcium oxalate and reduce the relative risk of stone [10]. Frequency of urination is usually enhanced by activity. Urgency is present in 40–50% of patients, and interruption of the stream in 30–40% of patients [11,12].

## 4 Conclusion

This rare case is, to the best of our knowledge, this is one of the largest bladder calcium oxalate stones case reported to date in China. For patients with only Lower urinary tract symptoms, bladder stone should be taken into consideration when other signs occur, such as recurrent urinary tract infection and hematuria. The combination of improved nutrition and modern antibiotic treatment has to be led to the frequency of bladder lithiasis. Calcium intake shouldn’t be restricted, whereas oxalate, sodium, and protein intakes have to be limited.

## Sources of funding

Nothing to declare.

## Ethical approval

This study is exempt from ethnical approval in our institution.

## Consent

Written informed consent was obtained from the patient for publication of this case report.

## Author contribution

Dr. Liu Yang Guang, Dr. Zhou Le, Dr. Hu Huan Chun: Performed the surgical technique, evaluation and post-operative manangement of the case.

Dr. Navin Shrestha: Drafting the article and final approval of the version to be submitted.

## Registration of research studies

N/A.

## Guarantor

Navin Shrestha

aecnavin@hotmail.com

## Provenance and peer review

Not commissioned, externally peer-reviewed.

## Declaration of Competing Interest

N/A.

## References

[1] Schwartz B.F., Stoller M.L. (2000). The vesical calculus. Urol. Clin. North Am..

[2] Childs M.A., Mynderse L.A., Rangel L.J., Wilson T.M., Lingeman J.E., Krambeck A.E. (2013). Pathogenesis of bladder calculi in the presence of urinary stasis. J. Urol..

[3] Bibilash B.S., Vijay A., Marickar Y.F. (2010). Stone composition and metabolic status. Urol. Res..

[4] Adam Childs M., Mynderse L.A., Rangel L.J. (2013). Pathogenesis of bladder calculi in the presence of urinary stasis. J. Urol..

[5] Agha R.A., Borrelli M.R., Farwana R., Koshy K., Fowler A., Orgill D.P., For the SCARE Group (2018). The SCARE 2018 Statement: updating consensus Surgical CAse REport (SCARE) Guidelines. Int. J. Surg..

[6] Mager R., Neisius A. (2019). Current concepts on the pathogenisis of urinary stones. Urologe A.

[7] Papatsoris A.G., Varkarakis I., Dellis A., Deliveliotis C. (2006). Bladder lithiasis: from open surgery to lithotripsy. Urol. Res..

[8] Farshi A., Sari Motlagh R., Jafari Arismani R. (2014). Delivery of huge bladder stone in a thirty-five-year-old man. Nephrourol. Mon..

[9] Peng X., Jifu Z., Wenwei C. (2014). A huge bladder calcium oxalate stone. Urolithiasis.

[10] Trinchieri A. (2013). Diet and renal stone formation. Minerva Med..

[11] Menon M., Resnick M.I., Walsh P.C., Retik A.B., Vaughan E.D., Wein A.J. (2002). Urinary lithiasis: etiology, diagnosis, and medical management. Campell’s Urology.

[12] Drach G.W., Walsh P.C., Retik A.B., Stamey T.A., Vaughan E.D. (1992). Urinary lithiasis: etiology, diagnosis, and medical management. Campell’s Urology.

